# DNA methylation of dopamine-related gene promoters is associated with line bisection deviation in healthy adults

**DOI:** 10.1038/s41598-019-42553-8

**Published:** 2019-04-11

**Authors:** Judith Schmitz, Robert Kumsta, Dirk Moser, Onur Güntürkün, Sebastian Ocklenburg

**Affiliations:** 1 Biopsychology, Institute of Cognitive Neuroscience, Department of Psychology, Ruhr University, Bochum, Germany; 2 Genetic Psychology, Department of Psychology, Ruhr University, Bochum, Germany

## Abstract

Handedness and language lateralization are the most investigated phenotypes among functional hemispheric asymmetries, i.e. differences in function between the left and the right half of the human brain. Both phenotypes are left hemisphere-dominant, while investigations of the molecular factors underlying right hemisphere-dominant phenotypes are less prominent. In the classical line bisection task, healthy subjects typically show a leftward attentional bias due to a relative dominance of the right hemisphere for visuospatial attention. Based on findings of variations in dopamine-related genes affecting performance in the line bisection task, we first tested whether DNA methylation in non-neuronal tissue in the promoter regions of *DBH*, *SLC6A3*, and *DRD2* are associated with line bisection deviation. We replicated the typical behavioral pattern and found an effect of DNA methylation in the *DBH* promoter region on line bisection deviation in right-aligned trials. A second exploratory analysis indicated that an overall DNA methylation profile of genes involved in dopamine function predicts line bisection performance in right-aligned trials. Genetic variation in dopamine-related genes has been linked to attention deficit hyperactivity disorder (ADHD), a neurodevelopmental trait associated with rightward attentional bias. Overall, our findings point towards epigenetic markers for functional hemispheric asymmetries in non-neuronal tissue not only for left hemisphere-dominant, but also for right hemisphere-dominant phenotypes.

## Introduction

Like most of our inner organs, human brain and behavior are asymmetrically organized^1^. However, in contrast to visceral asymmetry, hemispheric specialization for different cognitive and motor functions shows more interindividual variability, reflecting the immense complexity of brain asymmetries^2^. The ontogenesis of structural^3^ and functional hemispheric asymmetries such as handedness^4,5^ and language lateralization^6,7^ is partly influenced by genetic variation. However, in line with findings on environmental factors also playing a role, recent research has also suggested epigenetic regulation to contribute to the development of functional hemispheric asymmetries^8–10^. Epigenetic mechanisms summarize several chemical modifications to the DNA itself or proteins involved in DNA packaging that regulate the accessibility of transcription factors to the DNA, thus enhancing or repressing gene expression^11^. Empirical research on the epigenetics of hemispheric asymmetries so far mainly focused on left hemisphere-dominant phenotypes. For handedness, two studies have identified epigenetic markers in non-neuronal tissue^10,12^. For language lateralization, our recent work has revealed an effect of DNA methylation in the *KIAA0319* promoter region on attentional modulation of language lateralization, but not on language lateralization *per se*^13^. However, in order to come closer to a full understanding of hemispheric asymmetries, right hemisphere-dominant phenotypes also need to be considered.

**Figure 1:**
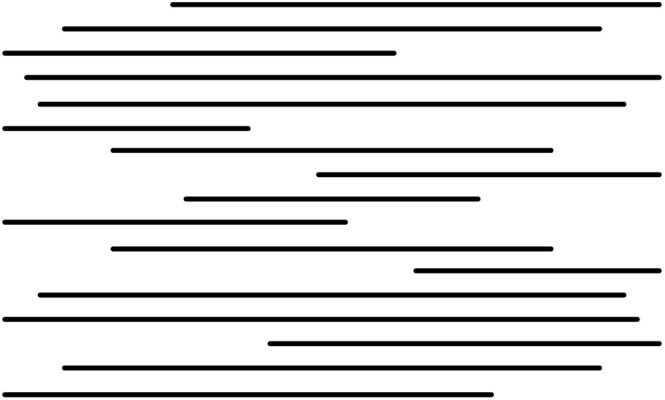
Illustration of the line bisection task.

One of the most investigated right hemisphere-dominant processes is visuospatial attention^14^. In the classical line bisection task (see Fig. 1), subjects are instructed to determine the center of a horizontal line^15,16^. While patients with right-hemispheric lesions tend to show a rightward bias due to neglect of the left hemifield^17^, healthy subjects typically show a leftward bias, a phenomenon that has been called pseudoneglect^18^. As the right hemisphere is typically dominant for visuospatial attention, the left hemifield is thought to be overrepresented, leading to a leftward bias^19^. In a meta-analysis of 73 studies in healthy subjects, a significant leftward bisection error was confirmed. The analysis also revealed that left-hand use results in stronger pseudoneglect than right-hand use (independent of handedness). Moreover, pseudoneglect is more pronounced in right-handers compared to left-handers (independent of hand use) and in men compared to women^20^.

Performance in the line bisection task is linked to brain structure and function. In line with lesion studies, fMRI studies in healthy subjects revealed activation in right-hemispheric posterior parietal areas during visuospatial attention tasks^14,21^. Correspondingly, rTMS over the right posterior parietal cortex induced a rightward shift in healthy subjects^22^. Using electrical stimulation during brain surgery, Thiebaut de Schotten *et al*.^23^ could show that the bisection error in the line bisection task depends on stimulation of the right-hemispheric inferior parietal lobule or the right-hemispheric caudal superior temporal gyrus. Due to an involvement of the superior occipitofrontal fasciculus, it was concluded that not only the parietal cortex, but also communication between the parietal and frontal cortex is crucial for visuospatial attention and neglect^23^. This conclusion was confirmed in a subsequent DTI study^24^.

Brain function likely acts as an intermediate phenotype between molecular determinants of functional hemispheric asymmetries and the observable phenotype^8^. Among genetic variations affecting attentional bias, several candidate genes affecting dopaminergic pathways have been investigated. This selection of candidate genes is based on a direct role of dopamine and noradrenaline in lateralized visuospatial attention that has been concluded from research in animals and humans. For example, unilateral injections of 6-hydroxydopamine induce lesions of the dopaminergic pathway, thereby causing spatial neglect in monkeys^25^ and rats^26^. In humans, dopamine agonists have shown beneficial effects on hemispatial neglect after right-hemispheric stroke^27^. Recently, it has been shown that other motivational factors such as monetary reward, which are also mediated by dopaminergic pathways, are also effective in reducing neglect after right-hemispheric stroke. Dopaminergic stimulation, however, is only effective in the absence of other motivational factors^28^. In healthy subjects, asymmetric binding of dopamine D2 receptors in striatal and cortical areas predicts visuospatial attention bias in the grayscales task^29^. Suppression or reversal of pseudoneglect has also been reported in normal aging and associated with age-related loss of dopamine^30^.

The dopamine beta-hydroxylase gene (*DBH*) encodes for the protein converting dopamine to norepinephrine^31^. In healthy adults, homozygous T allele carriers for the C-1021T polymorphism (linked to increased dopamine availability) have displayed an enhanced visuospatial attention bias towards the right side^32^. Similarly, homozygous carriers of the 9-repeat allele located in the 3′ untranslated region (3′UTR) of the dopamine transporter gene (*SLC6A3; DAT1*), also leading to increased availability of dopamine, resulted in greater rightward spatial bias^32^. In contrast, the 10-repeat allele has been linked to rightward bias in children affected by ADHD^33,34^. Moreover, homozygous carriers of the dopamine receptor D2 (*DRD2*) gene A2 allele showed significantly less rightward bias in a visuospatial attention task^35^

Overall, these findings suggest a genetic component in the ontogenesis of visuospatial attention bias. However, pseudoneglect has been shown to be modulated by different environmental factors. For example, reduced pseudoneglect has been reported in experienced videogame players^36^ or in urbanized compared to remote people^37^. Our first aim was to investigate potential peripheral epigenetic markers in the promoter regions of *DBH*, *SLC6A3*, and *DRD2* on the line bisection task in healthy adults. As genetic variation in these genes has been directly associated with this task, we chose a hypothesis-driven approach by only selecting these genes for analysis. We hypothesized that DNA methylation in the corresponding promoter regions predicts deviation from the midline in the line bisection task. In a second exploratory analysis, we assessed all genes involved in dopamine function to evaluate if a general DNA methylation profile predicts line bisection performance. As DNA methylation is tissue-specific, findings are interpreted as epigenetic signatures of visuospatial attention bias in non-neuronal tissue^38,39^.

## Results

**Figure 2:**
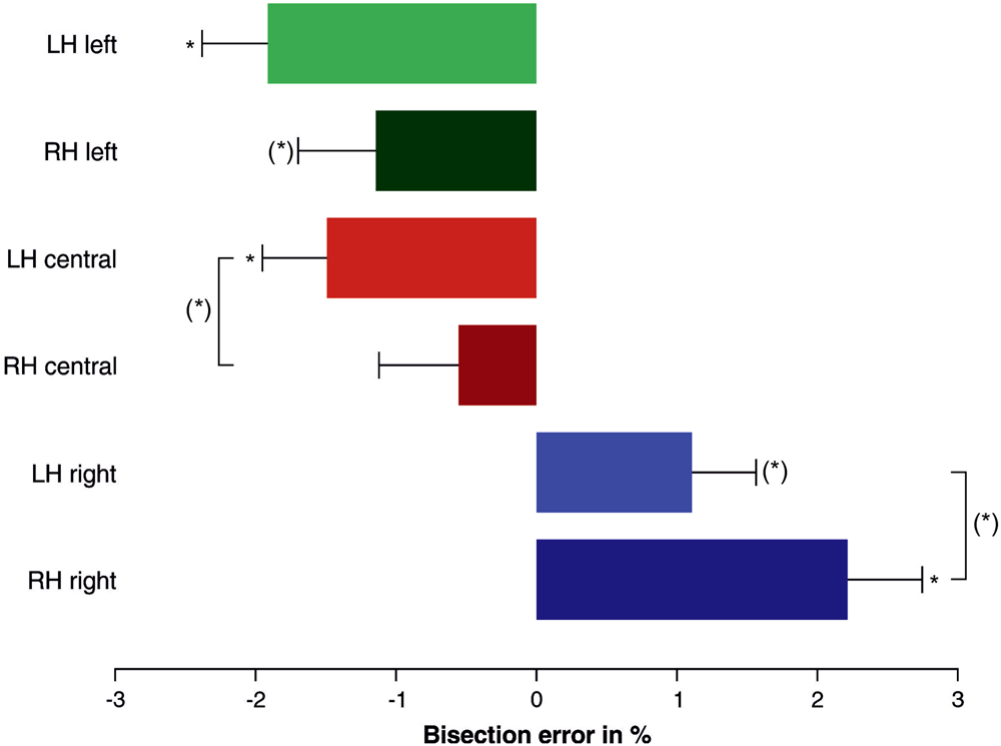
Bisection error in percent for left-aligned, central, and right-aligned trials performed with either the left hand (LH) or the right hand (RH). *p < 0.011, (*) nominal significance. Error bars indicate standard errors.

For the line bisection task, the repeated measures ANOVA revealed a significant main effect of condition (*F*(2,94) = 34.18, *p* = 0.000, partial η^2^ = 0.42) and a significant main effect of hand (*F*(1,47) = 6.04, *p* = 0.018, partial η^2^ = 0.11). No interaction with handedness or sex reached statistical significance. Bonferroni-corrected post-hoc tests revealed that subjects showed less pseudoneglect (a rightward bias) when lines were right-aligned, in contrast to the left-aligned (+3.16%, 95%-CI[1.95, 4.37], *p* = 0.000) and central trials (+2.68%, 95%-CI[1.79, 3.57], *p* = 0.000), which were not significantly different from each other (−0.48%, 95%-CI[−1.42, 0.46], *p* = 0.636). The main effect of hand was due to less pseudoneglect (a rightward bias) in right-handed compared to left-handed trials (+0.99%, 95%-CI[0.18, 1.80], *p* = 0.018). We performed one-sample t-tests against zero for each condition with Bonferroni correction for 6 comparisons (α = 0.011) to test for pseudoneglect. In line with previous studies^40^, we found significant pseudoneglect in left-aligned and central trials when using the left hand (left-aligned: *t*(50) = −4.10, *p* = 0.000, central: *t*(50) = −3.25, *p* = 0.002). However, t-tests were only nominally significant for left-aligned (*t*(50) = −2.07, *p* = 0.043) and non-significant for central trials performed with the right hand (*t*(50) = −0.98, *p* = 0.331). For right-aligned trials, there was significant rightward bias in right-handed trials (*t*(50) = 4.17, *p* = 0.000), but only nominally significant in left-handed trials (*t*(50) = 2.43, *p* = 0.019) (see Fig. 2). In order to test whether there was a significant difference between left-and right-handed trials in each condition, paired t-tests were performed with Bonferroni correction for 3 comparisons (α = 0.017). For left-aligned trials, pseudoneglect did not significantly differ between left- and right-handed trials (*t*(50) = −1.34, *p* = 0.186). For the other two conditions, there was a nominally significant difference with left-hand trials leading to more leftward bias than right-hand trials (central: *t*(50) = −2.10, *p* = 0.041; right-aligned: *t*(50) = −2.14, *p* = 0.037) (see Fig. 2).

**Figure 3:**
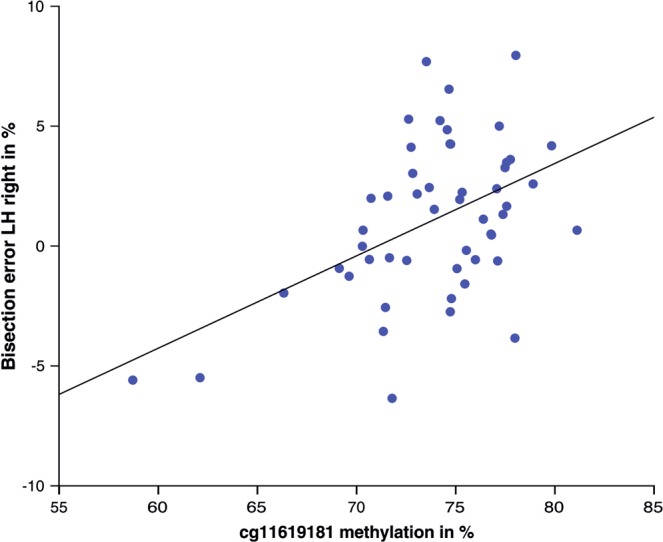
Scatterplot of DNA methylation of *DBH* cg11619181 in % and corresponding bisection error in the LH right condition in %.

In the hypothesis-driven analysis, DNA methylation in the *DRD2* and *SLC6A3* promoter regions did not significantly predict the percentage of line bisection deviation in any of the six conditions (all *p* > 0.0028). For *DBH*, the regression reached significance for the right-aligned trials performed with the left hand (*F*(1,49) = 14.88, *p* = 0.000, R^2^ = 0.22). The beta weight for one individual predictor reached significance (cg11619181: β = 0.48, *t* = 3.86, *p* = 0.000) (see Fig. 3). The regression was non-significant for the other conditions (all *p* > 0.0028).

In the exploratory analysis of DNA methylation, the regression did not reach significance for left-aligned and central trials performed with the left or right hand (all *p* > 0.0083). For right-aligned trials performed with the left hand, the regression model reached nominal significance (*F*(1,49) = 6.57, *p* = 0.014, R^2^ = 0.12). The individual beta weight reached nominal significance for PC1 (β = −0.34, *t* = −2.56, *p* = 0.014). For right-aligned trials performed with the right hand, the regression model reached significance (*F*(2,48) = 6.41, *p* = 0.003, R^2^ = 0.21). Individual beta weights reached nominal significance for sex (β = 0.33, *t* = 2.60, *p* = 0.012) and PC1 (β = −0.30, *t* = −2.30, *p* = 0.026).

## Discussion

The first aim of the present study was to investigate the effect of DNA methylation in the promoter regions of three dopamine candidate genes on attentional bias in the line bisection task. In previous studies conducted on buccal cells, we have shown an association of DNA methylation in promoter regions of respective candidate genes with handedness^12^ and attentional modulation of language lateralization^13^. Here, we aimed to test whether such an association could also be shown for a right hemisphere-dominant phenotype such as attentional bias. The second aim was to evaluate if an overall DNA methylation pattern in dopamine-related genes can predict deviation from the midline in the line bisection task.

The behavioral data are very much in line with what has been reported in studies with much larger sample sizes^20,40^, with a leftward bias in left-aligned and central trials, but a rightward bias in right-aligned trials. By trend, leftward bias was larger in trials performed with the left hand compared to the right. In the hypothesis-driven analysis, DNA methylation in the *DBH* promoter region significantly predicted line bisection deviation in right-aligned trials performed with the left hand. This effect was based on a single CpG site and independent from sex or handedness.

The *DBH* gene encodes for a protein converting dopamine to norepinephrine, thus strongly affecting dopamine availability. It has been linked to emotion processing, addiction^41^ and several neuropsychiatric disorders such as bipolar disorder^42^ and Parkinson’s disease^43^. Moreover, *DBH* might modulate psychotic symptoms^44^ and cognitive functioning in patients affected by schizophrenia^45^. In healthy subjects, the *DBH* genotype has been linked to cognition, especially attention^46^ and attentional bias for facial expression^47^. This is in line with genotype-associated corticostriatal-limbic activity revealed by fMRI^41^. To the best of our knowledge, only one study has been published on behavioral epigenetics of *DBH*. In an epigenome-wide DNA methylation study comparing alcohol dependent and control subjects, hypomethylation of CpG sites within *DBH* was associated alcohol dependence^48^. Alcoholism has also been linked to the development of hemispheric asymmetries as alcohol dependent patients seem to be more often non-right-handed than controls^49,50^. Moreover, alcoholism was associated to impairment of right hemisphere function in cued detection^51^ and visuospatial tasks^52,53^. In contrast, acute alcohol consumption leads to a more pronounced leftward bias in the line bisection task^54^. In search of potential environmental factors influencing DNA methylation of the *DBH* promoter, the only study in rats hints towards altered transcription levels of *DBH* through stress induced by sleep deprivation^55^.

In the exploratory analysis, DNA methylation in promoter regions of dopamine-related genes was associated with performance in the line bisection task in right-aligned trials performed with the right hand and by trend with the left hand. This analysis is in line with dopamine-related genes playing a role in visuospatial attentional bias, not only at the level of genetic variation. The balance of dopamine and noradrenaline plays an important role in the etiology of attention deficit hyperactivity disorder (ADHD), making *DBH*^56^ and other dopamine-relevant genes^57^ important influencing factors in the development of ADHD. In rats, 6-hydroxydopamine injection not only disrupts the dopaminergic pathway and induces neglect^26^, but also ADHD-like behavior^58^. Interestingly, several studies have revealed a rightward bias in the line bisection task in ADHD patients^59,60^ and healthy subjects with ADHD-like behavior^61^. This is in line with the idea that ADHD is associated with right hemisphere inefficiency and dysfunction^62^ that is also reflected in atypical asymmetry in the frontostriatal network^63,64^. There are several prenatal risk factors to the development of ADHD that might be mediated by epigenetic regulation^65^. Using cord blood at birth, an epigenome-wide association study on ADHD symptoms recently revealed 13 CpG sites showing differential DNA methylation between different trajectories of ADHD symptoms^66^. Peripheral epigenetic markers of ADHD have also been reported in an epigenome-wide association study on salivary DNA^67^.

In laterality research, the most investigated phenotypes are handedness and language lateralization, which are both left hemisphere-dominant in the majority of individuals^68^. The present study suggests peripheral epigenetic markers of visuospatial attentional bias, which is usually right hemisphere-dominant^69^, leading to a complementary specialization for language and visuospatial attention in the majority of individuals. Moreover, subjects showing right hemisphere dominance for language have left hemisphere dominance for visuospatial attention, suggesting a causal relationship with hemispheric specialization of one function determining the other^70^. This finding mostly contradicts early single gene theories^71^ which assumed that lateralization of different functions is independent from each other^70^. However, molecular factors associated with both left and right hemisphere-dominant functions remain to be uncovered.

In conclusion, our data suggest that DNA methylation in the promoter region of the *DBH* gene and other dopamine-related genes is associated with attentional bias in the line bisection task, a right hemisphere-dominant phenotype. The obvious limitation of this study is that DNA was extracted from buccal cells instead of brain tissue. However, it has been suggested that interindividual variation of cerebral DNA methylation is reflected in peripheral tissue^72^. Thus, the findings from the present study should be interpreted cautiously as peripheral epigenetic markers of visuospatial attention bias. Midline deviation in the line bisection task is likely influenced by multiple genetic, epigenetic, and environmental factors that are not uncovered in the present study and will be determined in future large-scale studies. Future research should also integrate epigenetic markers on laterality phenotypes with brain structure and function. Moreover, replication in samples selected for ADHD or other traitsassociated with a rightward bias of attention such as obsessive compulsive disorders (OCD)^73^ or personality disorders^74^ might be worthwhile.

## Methods

### Subjects

Fifty-one (25 female) healthy German university students between 19 and 33 years of age (mean 24.41 years, SD 3.01 years) participated in the study. Handedness was determined using the Edinburgh handedness inventory (EHI)^75^. Since sex and handedness might have an effect on line bisection performance^20^, we balanced men and women as well as left- and right-handers in our sample. Among all subjects, 24 (11 females) were consistently left-handed as indicated by an EHI LQ < −60 and 27 (14 females) were consistently right-handed as indicated by an EHI LQ > 60. All subjects had normal or corrected to normal vision and had no history of neurological or psychiatric disease. All subjects gave written informed consent and were treated in accordance with the declaration of Helsinki. The study was approved by the ethics committee of the Psychological Faculty at Ruhr University Bochum.

### Behavioral assessment

The line bisection task was used to determine laterality of visuospatial attention similar to previous studies^40,76^. Seventeen horizontal lines of 10.0 to 26.0 cm in length and 1 mm in width were presented on a white sheet of paper with 21.0 cm × 29.7 cm in size that was centered on the table in front of the subject. Five horizontal lines were positioned left-aligned, seven were positioned centrally and five were positioned right-aligned (see Fig. 1). Subjects were asked to bisect each line at the midpoint with a fine pencil. The task was completed with the left and the right hand each with the order balanced across subjects. Overall, this procedure resulted in six conditions depending on the position of the line (left, central, right) and the hand used to complete the task (left, right).

### DNA methylation

DNA was isolated from buccal cells that were brushed from the subjects’ oral mucosa using buccal swabs. DNA isolation was conducted with the blackPREP Swab DNA Kit (Analytik Jena, Germany). The isolated gDNA was stored at −20 °C. The EpiTect Kit (Qiagen, Germany) was used for bisulfite conversion of 500 ng gDNA. After elution of bisulfite-converted DNA in 10 µl elution buffer, 4 µl were used for analysis on the MethylationEPIC array (Illumina, CA, USA).

### Bioinformatics

Preprocessing and processing of the data was performed using RStudio^77^ version 0.99.903 and the Bioconductor packages implemented in the RnBeads workflow^78^. Overall, 867,926 probes were imported from signal intensity data (idat). During quality control, we excluded potential sample mix-ups or duplications, unspecific probe hybridization and problems with bisulfite conversion. Probes were removed in cases of high detection *p* values (>0.01) or overlap with known SNPs. Moreover, probes showing unreliable measurements (using the Greedycut algorithm), non-CpG (CpH) probes and gonosomal probes were removed from the dataset. The methylation ß values of the remaining probes were normalized using the β-mixture quantile (BMIQ) method^79^ and annotated using the reference genome GRCh37 (hg19). Promoter regions were defined as 1500 bp upstream and 500 bp downstream of transcription start sites.

Table 1 shows the location of promoter regions and number of included CpG sites for the candidate genes *DBH* (chr9: 136501482-136524466), *SLC6A3* (chr5: 1392909-1445545), and *DRD2* (chr11: 113284586-113346413). No effect of genetic imprinting was found in the current literature for *DRD2* or *DBH*. However, there is evidence for paternal expression of *SLC6A3*^80,81^.

**Table 1:** Promoter regions of examined genes with chromosomal locations and number of tested CpG sites.

Gene	Chromosome	Start of promoter region	End of promoter region	Number of CpG sites tested within the promoter region
*DBH*	9	136499982	136501981	8
*SLC6A3*	5	1445046	1447045	9
*DRD2*	11	113345914	113347913	10

In order to analyze a possible association between line bisection performance and DNA methylation in promoter regions of dopamine-related genes, we performed a search for all genes involved in dopamine function using the Gene Ontology website using the term “dopamine” (http://geneontology.org/). After filtering for *homo sapiens*, this tool revealed 186 dopamine-related genes, 171 of which were also available on the MethylationEPIC array after preprocessing. The genes as well as chromosomal positions of the corresponding promoter regions are found in Supplementary Table S1.

### Statistical analysis

For the line bisection task, the percentage of deviation from the midline of each horizontal line was determined using the formula [(measured left half − true half) / true half] × 100, which resulted in negative values indicating a leftward bias and positive values indicating a rightward bias. A mean score was calculated for each condition and each hand used. The effect of condition and hand was calculated using a 3 × 2 repeated measures ANOVA with the within-subject factor condition (left-aligned, centered, right-aligned) and hand (left, right) and the between-subject factors sex and handedness. For the ANOVA, partial η^2^ is reported as a measure of effect size. Bonferroni-adjusted post hoc tests were performed for significant main and interaction effects.

#### Hypothesis-driven analysis of DNA methylation

The hypothesis-driven analysis was aimed at evaluating if DNA methylation in promoter regions of candidate genes are associated with line bisection performance at single CpG site level. For each of the 6 conditions and each of the 3 genes, we conducted a linear step-wise regression analysis with individual % DNA methylation levels of all CpG sites within the respective promoter region, sex and handedness as predictors and the percentage of line bisection deviation as dependent variable. To correct for multiple comparisons, the alpha level was set to 0.0028 after Bonferroni correction (0.05 / 3 genes / 6 conditions = 0.0028). R^2^ is reported as a measure of effect size.

#### Exploratory analysis *of DNA methylation*

The exploratory analysis was aimed at evaluating if an overall DNA methylation profile predicts line bisection performance at the level of promoter regions. As DNA methylation levels are intercorrelated and to reduce the number of predictors, principal component analysis (PCA) was performed on mean % DNA methylation levels of the promoter regions of the 171 dopamine-related genes^10^. PCA revealed 28 principal components (PCs) with an eigenvalue >1. The first four PCs (PC1, PC2, PC3 and PC4) were considered for further analysis as determined per screeplot. Factor loadings are shown in Supplementary Table S2. *DRD4* showed no factor loading >0.2 with any of the PCs and was excluded from analysis. PCA was repeated without *DRD4*. PC1, PC2, PC3, PC4, sex and handedness were used as predictors in a linear step-wise regression analysis using the percentage of line bisection deviation as dependent variable. This analysis was performed for each of the 6 conditions. To correct for multiple comparisons, the alpha level was set to 0.0083 after Bonferroni correction (0.05 / 6 conditions = 0.0083). R^2^ is reported as a measure of effect size.

ANOVAs, PCA and linear regression analyses were calculated using IBM SPSS Statistics 20 (IBM, United States).

## Supplementary information

Supplementary Information

## Data Availability

The dataset generated during the current study is available from the corresponding author on request.
