# Supplementary material for: DNA methylation of dopamine-related gene promoters is associated with line bisection deviation in healthy adults
Source: Sci Rep. 2019 Apr 11;9:5902. doi: 10.1038/s41598-019-42553-8 (PMC6459813; doi:10.1038/s41598-019-42553-8)
Supplement: Supplementary file 1 — Supplementary Information [file 41598_2019_42553_MOESM1_ESM.pdf]

**Supplementary information**

**DNA methylation of dopamine-related gene promoters is associated with line bisection deviation in healthy adults**

Judith Schmitz, Robert Kumsta, Dirk Moser, Onur Güntürkün, Sebastian Ocklenburg

**Table S1: Dopamine-related genes included in the exploratory analysis.**

| **Chromosome** | **Symbol** | **Start of promoter region** | **End of promoter region** |
|---|---|---|---|
| chr1 | *CHRNB2* | 154538757 | 154540756 |
| chr1 | *DMRTA2* | 50888673 | 50890672 |
| chr1 | *GNAI3* | 110089733 | 110091732 |
| chr1 | *GNB1* | 1821996 | 1823995 |
| chr1 | *HCN3* | 155245874 | 155247873 |
| chr1 | *KCNA2* | 111173597 | 111175596 |
| chr1 | *LMX1A* | 165325453 | 165327452 |
| chr1 | *NCSTN* | 160311562 | 160313561 |
| chr1 | *NPR1* | 153649613 | 153651612 |
| chr1 | *PARK7* | 8012851 | 8014850 |
| chr1 | *PINK1* | 20958448 | 20960447 |
| chr1 | *PTGS2* | 186649060 | 186651059 |
| chr1 | *RAB3B* | 52455937 | 52457936 |
| chr1 | *RGS4* | 163037065 | 163039064 |
| chr1 | *RGS8* | 182653212 | 182655211 |
| chr1 | *SYT11* | 155827800 | 155829799 |
| chr1 | *SYT2* | 202679046 | 202681045 |
| chr1 | *SYT6* | 114696042 | 114698041 |
| chr1 | *TGFB2* | 218518077 | 218520076 |
| chr1 | *VANGL2* | 160368876 | 160370875 |
| chr1 | *WNT3A* | 228193252 | 228195251 |
| chr2 | *ALK* | 30143933 | 30145932 |
| chr2 | *EN1* | 119604755 | 119606754 |
| chr2 | *NR4A2* | 157198361 | 157200360 |
| chr2 | *PNKD* | 219133615 | 219135614 |
| chr2 | *PPP1R1C* | 182817468 | 182819467 |
| chr3 | *ADCY5* | 123168106 | 123170105 |
| chr3 | *CELSR3* | 48699849 | 48701848 |
| chr3 | *CTNNB1* | 41234828 | 41236827 |
| chr3 | *DRD3* | 113917755 | 113919754 |
| chr3 | *GSK3B* | 119812765 | 119814764 |
| chr3 | *MANF* | 51420978 | 51422977 |
| chr3 | *RYK* | 133969190 | 133971189 |
| chr3 | *WNT5A* | 55523474 | 55525473 |

| | | | |
|---|---|---|---|
| chr4 | *DRD5* | 9781758 | 9783757 |
| chr4 | *NPY2R* | 156128281 | 156130280 |
| chr4 | *PHOX2B* | 41750488 | 41752487 |
| chr4 | *SFRP2* | 154709773 | 154711772 |
| chr4 | *SNCA* | 90758967 | 90760966 |
| chr4 | *TACR3* | 104640474 | 104642473 |
| chr5 | *CRHBP* | 76247038 | 76249037 |
| chr5 | *DRD1* | 174870712 | 174872711 |
| chr5 | *GDNF* | 37839289 | 37841288 |
| chr5 | *HTR1A* | 63257835 | 63259834 |
| chr5 | *SLC6A3* | 1445046 | 1447045 |
| chr5 | *SNCAIP* | 121645549 | 121647548 |
| chr5 | *SNCB* | 176057031 | 176059030 |
| chr6 | *CNR1* | 88875579 | 88877578 |
| chr6 | *DTNBP1* | 15662790 | 15664789 |
| chr6 | *FLOT1* | 30710011 | 30712010 |
| chr6 | *HDAC2* | 114331973 | 114333972 |
| chr6 | *HTR1B* | 78172991 | 78174990 |
| chr6 | *MOXD1* | 132722185 | 132724184 |
| chr6 | *OPRM1* | 154330131 | 154332130 |
| chr6 | *SLC22A1* | 160541321 | 160543320 |
| chr6 | *SLC22A3* | 160767800 | 160769799 |
| chr6 | *VEGFA* | 43736421 | 43738420 |
| chr7 | *CAV2* | 115925934 | 115927933 |
| chr7 | *CDK5* | 150755118 | 150757117 |
| chr7 | *DDC* | 50632655 | 50634654 |
| chr7 | *FZD1* | 90892283 | 90894282 |
| chr7 | *GNA12* | 2883459 | 2885458 |
| chr7 | *GPR37* | 124405182 | 124407181 |
| chr7 | *MOXD2P* | 141946387 | 141948386 |
| chr7 | *SHH* | 155604468 | 155606467 |
| chr7 | *WNT2* | 116962844 | 116964843 |
| chr8 | *ADRA1A* | 26724291 | 26726290 |
| chr8 | *CHRNB3* | 42551019 | 42553018 |
| chr8 | *CRH* | 67090461 | 67092460 |
| chr8 | *FGF20* | 16859191 | 16861190 |
| chr8 | *FZD3* | 28350229 | 28352228 |
| chr8 | *MAPK15* | 144796929 | 144798928 |
| chr8 | *OPRK1* | 54163758 | 54165757 |
| chr8 | *RSPO2* | 109095414 | 109097413 |
| chr8 | *SFRP1* | 41166517 | 41168516 |
| chr9 | *ABL1* | 133587833 | 133589832 |
| chr9 | *DBH* | 136499982 | 136501981 |
| chr9 | *DNM1* | 130964158 | 130966157 |
| chr9 | *GNA14* | 80262724 | 80264723 |
| chr9 | *LMX1B* | 129375222 | 129377221 |

| | | | |
|---|---|---|---|
| chr9 | *OR13F1* | 107264955 | 107266954 |
| chr9 | *TOR1A* | 132585914 | 132587913 |
| chr10 | *CALY* | 135149976 | 135151975 |
| chr10 | *CDNF* | 14880075 | 14882074 |
| chr10 | *CXCL12* | 44881442 | 44883441 |
| chr10 | *DKK1* | 54072556 | 54074555 |
| chr10 | *FGF8* | 103535355 | 103537354 |
| chr10 | *PITX3* | 104000732 | 104002731 |
| chr10 | *SLC18A2* | 118999104 | 119001103 |
| chr10 | *SNCG* | 88716875 | 88718874 |
| chr10 | *SYT15* | 46970901 | 46972900 |
| chr11 | *DRD2* | 113345914 | 113347913 |
| chr11 | *DRD4* | 635793 | 637792 |
| chr11 | *LRTOMT* | 71789882 | 71791881 |
| chr11 | *OR56A1* | 6048472 | 6050471 |
| chr11 | *OR56A4* | 6023925 | 6025924 |
| chr11 | *OR5T1* | 56041529 | 56043528 |
| chr11 | *PHOX2A* | 71956209 | 71958208 |
| chr11 | *SYT12* | 66772749 | 66774748 |
| chr11 | *SYT13* | 45307371 | 45309370 |
| chr11 | *SYT7* | 61348121 | 61350120 |
| chr11 | *SYT8* | 1847209 | 1849208 |
| chr11 | *SYT9* | 7258509 | 7260508 |
| chr11 | *TH* | 2192608 | 2194607 |
| chr12 | *ADCY6* | 49182321 | 49184320 |
| chr12 | *DAO* | 109251208 | 109253207 |
| chr12 | *LRP6* | 12419447 | 12421446 |
| chr12 | *PDE1B* | 54941634 | 54943633 |
| chr12 | *PPP1R1A* | 54981944 | 54983943 |
| chr12 | *PTPN11* | 112854655 | 112856654 |
| chr12 | *SYT1* | 79256273 | 79258272 |
| chr12 | *SYT10* | 33592255 | 33594254 |
| chr12 | *WNT1* | 49370898 | 49372897 |
| chr13 | *HTR2A* | 47470670 | 47472669 |
| chr14 | *FOXA1* | 38068746 | 38070745 |
| chr14 | *GCH1* | 55369071 | 55371070 |
| chr14 | *GNG2* | 52291413 | 52293412 |
| chr14 | *HIF1A* | 62160731 | 62162730 |
| chr14 | *OTX2* | 57276698 | 57278697 |
| chr14 | *PRMT5* | 23398295 | 23400294 |
| chr15 | *CHRM5* | 34259421 | 34261420 |
| chr15 | *CHRNA5* | 78856362 | 78858361 |
| chr15 | *GNB5* | 52483067 | 52485066 |
| chr15 | *SIN3A* | 75747684 | 75749683 |
| chr16 | *ABAT* | 8766922 | 8768921 |
| chr16 | *CNTNAP4* | 76309676 | 76311675 |

| | | | |
|---|---|---|---|
| chr16 | *FUK* | 70486824 | 70488823 |
| chr16 | *GNAO1* | 56223802 | 56225801 |
| chr16 | *GRIN2A* | 10276112 | 10278111 |
| chr16 | *ITGAM* | 31269811 | 31271810 |
| chr16 | *MAPK3* | 30134328 | 30136327 |
| chr16 | *SLC6A2* | 55688016 | 55690015 |
| chr16 | *SULT1A3* | 30204254 | 30206253 |
| chr16 | *SYT17* | 19177793 | 19179792 |
| chr16 | *VPS35* | 46722931 | 46724930 |
| chr17 | *ARRB2* | 4612284 | 4614283 |
| chr17 | *CSNK1D* | 80231108 | 80233107 |
| chr17 | *DLG4* | 7122522 | 7124521 |
| chr17 | *GNA13* | 63052458 | 63054457 |
| chr17 | *NLGN2* | 7306693 | 7308692 |
| chr17 | *PPP1R1B* | 37781493 | 37783492 |
| chr17 | *RGS9* | 63132049 | 63134048 |
| chr17 | *SLC6A4* | 28562521 | 28564520 |
| chr17 | *SLC9A3R1* | 72743291 | 72745290 |
| chr17 | *WNT3* | 44910021 | 44912020 |
| chr17 | *WNT9B* | 44909067 | 44911066 |
| chr18 | *SYT4* | 40857116 | 40859115 |
| chr19 | *DNM2* | 10827255 | 10829254 |
| chr19 | *GNA11* | 3092908 | 3094907 |
| chr19 | *GNA15* | 3134691 | 3136690 |
| chr19 | *GSK3A* | 42746278 | 42748277 |
| chr19 | *KLF16* | 1863068 | 1865067 |
| chr19 | *PALM* | 707453 | 709452 |
| chr19 | *PTGER1* | 14585675 | 14587674 |
| chr19 | *SYT3* | 51171152 | 51173151 |
| chr19 | *SYT5* | 55691310 | 55693309 |
| chr20 | *CHRNA4* | 62009254 | 62011253 |
| chr20 | *FOXA2* | 22565594 | 22567593 |
| chr20 | *GNAS* | 57413273 | 57415272 |
| chr20 | *ID1* | 30191586 | 30193585 |
| chr20 | *SNX5* | 17949124 | 17951123 |
| chr21 | *CLIC6* | 36040188 | 36042187 |
| chr21 | *GABPA* | 27105381 | 27107380 |
| chr21 | *ITGB2* | 46351405 | 46353404 |
| chr21 | *TIAM1* | 32931791 | 32933790 |
| chr22 | *ADORA2A* | 24812347 | 24814346 |
| chr22 | *ATF4* | 39914200 | 39916199 |
| chr22 | *COMT* | 19927630 | 19929629 |
| chr22 | *CSNK1E* | 38794028 | 38796027 |
| chr22 | *MAPK1* | 22221471 | 22223470 |
| chr22 | *RASD2* | 35935415 | 35937414 |

**Table S2: Factor loadings from PCA for dopamine-related genes.**

| Gene | PC1 loading | PC2 loading | PC3 loading | PC4 loading |
|---|---|---|---|---|
| *ITGAM* | -0,96 | 0,08 | -0,20 | 0,10 |
| *SFRP1* | -0,95 | 0,13 | -0,08 | -0,04 |
| *SFRP2* | -0,94 | 0,03 | -0,11 | 0,06 |
| *CRHBP* | -0,93 | 0,11 | -0,17 | 0,14 |
| *LMX1A* | -0,92 | 0,12 | -0,18 | -0,06 |
| *RGS8* | -0,92 | -0,03 | -0,17 | -0,02 |
| *LRTOMT* | 0,92 | 0,19 | 0,24 | 0,14 |
| *WNT3* | 0,90 | 0,25 | 0,20 | 0,19 |
| *KCNA2* | -0,90 | 0,19 | -0,19 | 0,17 |
| *CHRNA5* | -0,90 | 0,24 | -0,04 | 0,05 |
| *FGF20* | 0,89 | 0,21 | 0,28 | 0,16 |
| *GNB5* | -0,89 | 0,12 | -0,33 | 0,12 |
| *WNT9B* | 0,88 | 0,23 | 0,23 | 0,20 |
| *PPP1R1B* | 0,86 | 0,29 | 0,31 | 0,16 |
| *VANGL2* | 0,86 | 0,23 | 0,36 | 0,06 |
| *MAPK15* | 0,84 | 0,32 | 0,09 | 0,12 |
| *GNA13* | 0,83 | 0,28 | 0,35 | 0,20 |
| *SYT8* | 0,83 | 0,29 | 0,21 | 0,25 |
| *MAPK3* | 0,83 | 0,15 | 0,46 | 0,11 |
| *PINK1* | 0,82 | 0,40 | 0,14 | 0,19 |
| *PTPN11* | 0,77 | 0,34 | 0,19 | 0,26 |
| *CLIC6* | -0,76 | 0,19 | -0,02 | 0,07 |
| *OR56A4* | 0,73 | 0,21 | 0,09 | 0,41 |
| *CHRNA4* | 0,72 | 0,45 | 0,31 | 0,21 |
| *PTGS2* | 0,70 | 0,34 | 0,25 | 0,25 |
| *GCH1* | -0,69 | 0,34 | -0,18 | 0,07 |
| *SLC22A3* | -0,68 | 0,37 | -0,05 | 0,02 |
| *PALM* | 0,68 | 0,31 | 0,31 | 0,29 |
| *SLC18A2* | 0,68 | 0,54 | 0,29 | 0,14 |
| *CHRM5* | 0,67 | 0,52 | 0,23 | -0,11 |
| *SYT2* | 0,67 | 0,34 | 0,51 | 0,08 |
| *SNCAIP* | -0,66 | 0,41 | 0,23 | 0,27 |
| *SLC9A3R1* | 0,65 | 0,39 | 0,54 | 0,00 |
| *CSNK1D* | 0,64 | 0,41 | 0,51 | 0,19 |
| *RGS9* | 0,63 | 0,50 | 0,29 | 0,29 |
| *WNT3A* | -0,63 | 0,26 | -0,10 | -0,03 |
| *CAV2* | -0,63 | 0,08 | -0,32 | 0,28 |
| *SLC6A2* | -0,63 | 0,62 | -0,20 | 0,06 |
| *TH* | 0,63 | 0,38 | 0,26 | 0,09 |
| *SYT7* | 0,63 | 0,49 | 0,08 | 0,38 |
| *DAO* | -0,63 | 0,57 | 0,24 | 0,10 |
| *RGS4* | 0,60 | 0,30 | 0,29 | 0,07 |
| *GNA14* | -0,60 | 0,51 | -0,01 | 0,24 |

| | | | | |
|---|---|---|---|---|
| *TOR1A* | 0,57 | 0,12 | 0,40 | 0,20 |
| *OR13F1* | 0,56 | 0,31 | -0,24 | 0,26 |
| *NCSTN* | 0,55 | 0,33 | 0,53 | 0,21 |
| *GPR37* | 0,54 | 0,53 | 0,32 | 0,13 |
| *PNKD* | 0,53 | 0,48 | 0,42 | 0,32 |
| *GNA15* | 0,53 | 0,28 | 0,46 | 0,11 |
| *CDNF* | 0,52 | 0,30 | 0,43 | 0,25 |
| *SYT5* | -0,52 | 0,52 | 0,15 | 0,08 |
| *SYT15* | 0,52 | 0,47 | 0,14 | -0,19 |
| *DBH* | 0,51 | 0,32 | 0,23 | 0,46 |
| *SYT12* | 0,37 | 0,20 | 0,07 | 0,08 |
| *CHRNB3* | -0,35 | 0,24 | -0,20 | 0,26 |
| *MOXD2P* | -0,31 | -0,22 | -0,23 | 0,16 |
| *HTR1B* | 0,25 | 0,83 | 0,21 | 0,06 |
| *PHOX2A* | -0,13 | 0,79 | 0,05 | 0,07 |
| *PPP1R1C* | -0,27 | 0,79 | 0,27 | 0,02 |
| *PHOX2B* | 0,17 | 0,79 | 0,25 | 0,15 |
| *SYT1* | -0,16 | 0,79 | 0,16 | -0,05 |
| *DKK1* | -0,22 | 0,79 | 0,22 | 0,08 |
| *HTR1A* | 0,18 | 0,79 | 0,36 | -0,08 |
| *OTX2* | -0,46 | 0,78 | 0,07 | 0,07 |
| *EN1* | 0,15 | 0,76 | 0,27 | 0,08 |
| *PPP1R1A* | -0,33 | 0,76 | 0,21 | 0,31 |
| *SHH* | 0,28 | 0,76 | 0,29 | -0,02 |
| *DRD1* | 0,42 | 0,74 | 0,16 | 0,18 |
| *SYT17* | -0,02 | 0,73 | -0,02 | 0,34 |
| *DRD5* | 0,02 | 0,73 | -0,03 | 0,04 |
| *NPY2R* | 0,21 | 0,72 | 0,32 | -0,07 |
| *FOXA1* | 0,03 | 0,71 | 0,27 | 0,24 |
| *FOXA2* | 0,11 | 0,71 | 0,06 | -0,19 |
| *ABL1* | 0,20 | 0,71 | 0,37 | 0,08 |
| *CHRNB2* | 0,37 | 0,69 | 0,19 | 0,23 |
| *DRD2* | 0,40 | 0,69 | 0,28 | 0,22 |
| *SYT13* | 0,05 | 0,67 | 0,43 | 0,38 |
| *WNT2* | 0,28 | 0,67 | 0,07 | 0,33 |
| *CNR1* | 0,32 | 0,66 | 0,32 | 0,13 |
| *WNT1* | -0,33 | 0,65 | -0,01 | 0,36 |
| *SNCB* | -0,21 | 0,65 | 0,24 | 0,30 |
| *ATF4* | 0,05 | 0,65 | 0,14 | 0,30 |
| *ALK* | 0,17 | 0,65 | 0,22 | 0,28 |
| *SYT3* | 0,19 | 0,64 | 0,27 | 0,00 |
| *RSPO2* | 0,34 | 0,64 | 0,22 | 0,01 |
| *SNCG* | 0,01 | 0,61 | -0,04 | 0,40 |
| *DMRTA2* | 0,18 | 0,60 | 0,14 | 0,19 |
| *SYT6* | 0,12 | 0,59 | 0,17 | 0,18 |
| *HIF1A* | -0,24 | 0,58 | 0,41 | 0,19 |

| | | | | |
|---|---|---|---|---|
| *DNM1* | 0,39 | 0,57 | 0,20 | 0,49 |
| *PITX3* | -0,01 | 0,56 | 0,53 | 0,04 |
| *TIAM1* | 0,46 | 0,56 | 0,46 | 0,29 |
| *NR4A2* | 0,25 | 0,55 | 0,26 | 0,19 |
| *WNT5A* | 0,41 | 0,55 | 0,24 | 0,48 |
| *ADORA2A* | 0,51 | 0,55 | -0,18 | 0,28 |
| *TACR3* | 0,13 | 0,54 | 0,04 | 0,07 |
| *RAB3B* | -0,01 | 0,53 | 0,40 | 0,28 |
| *GNA11* | 0,51 | 0,52 | 0,21 | 0,14 |
| *SULT1A3* | 0,16 | 0,51 | 0,15 | 0,36 |
| *SYT10* | 0,20 | 0,50 | 0,41 | 0,03 |
| *FGF8* | 0,11 | 0,50 | 0,26 | 0,22 |
| *CRH* | 0,08 | 0,50 | -0,37 | 0,21 |
| *HCN3* | -0,07 | 0,47 | 0,29 | 0,14 |
| *COMT* | 0,10 | 0,45 | 0,26 | 0,31 |
| *CNTNAP4* | 0,19 | 0,44 | -0,44 | 0,36 |
| *PTGER1* | 0,33 | 0,42 | 0,13 | 0,35 |
| *PDE1B* | 0,04 | 0,40 | 0,25 | 0,35 |
| *OR56A1* | 0,38 | 0,39 | -0,01 | 0,35 |
| *CTNNB1* | 0,14 | 0,33 | -0,04 | 0,24 |
| *DTNBP1* | 0,07 | 0,32 | 0,25 | 0,19 |
| *HDAC2* | -0,22 | 0,22 | -0,16 | 0,06 |
| *FLOT1* | 0,21 | 0,27 | 0,87 | 0,11 |
| *ADCY6* | 0,12 | 0,06 | 0,86 | -0,14 |
| *MANF* | 0,20 | 0,03 | 0,85 | 0,09 |
| *GNB1* | 0,13 | 0,15 | 0,83 | -0,13 |
| *SIN3A* | 0,24 | -0,25 | 0,83 | -0,15 |
| *KLF16* | 0,07 | 0,02 | 0,80 | -0,14 |
| *GNAI3* | 0,18 | 0,04 | 0,80 | 0,09 |
| *ARRB2* | 0,19 | 0,13 | 0,79 | -0,02 |
| *GNAO1* | 0,35 | 0,12 | 0,77 | -0,09 |
| *FZD1* | 0,20 | -0,06 | 0,75 | 0,02 |
| *CDK5* | 0,45 | 0,37 | 0,73 | 0,11 |
| *LRP6* | 0,21 | 0,13 | 0,73 | 0,04 |
| *GSK3B* | 0,26 | 0,28 | 0,73 | 0,05 |
| *SNX5* | 0,35 | 0,25 | 0,72 | 0,30 |
| *NPR1* | 0,38 | 0,35 | 0,72 | 0,09 |
| *GNA12* | 0,21 | 0,16 | 0,72 | 0,11 |
| *GSK3A* | 0,15 | 0,38 | 0,71 | 0,08 |
| *ID1* | 0,16 | 0,38 | 0,69 | 0,14 |
| *TGFB2* | 0,27 | 0,28 | 0,69 | 0,33 |
| *MAPK1* | 0,64 | 0,14 | 0,67 | -0,05 |
| *LMX1B* | 0,36 | 0,07 | 0,67 | -0,36 |
| *FUK* | 0,33 | 0,11 | 0,65 | -0,02 |
| *CSNK1E* | 0,28 | 0,25 | 0,63 | 0,18 |
| *RASD2* | 0,39 | 0,52 | 0,62 | 0,14 |

| | | | | |
|---|---|---|---|---|
| *SLC6A4* | 0,34 | 0,43 | 0,62 | -0,02 |
| *CALY* | 0,20 | 0,10 | 0,61 | -0,38 |
| *GDNF* | 0,52 | 0,09 | 0,61 | 0,19 |
| *VPS35* | 0,17 | 0,51 | 0,61 | 0,24 |
| *ABAT* | -0,17 | 0,06 | 0,60 | 0,00 |
| *PRMT5* | 0,05 | 0,28 | 0,60 | -0,06 |
| *GABPA* | 0,54 | 0,24 | 0,60 | -0,02 |
| *GRIN2A* | 0,08 | 0,27 | 0,57 | -0,09 |
| *VEGFA* | 0,29 | 0,22 | 0,56 | 0,56 |
| *ADCY5* | 0,02 | 0,37 | 0,55 | 0,35 |
| *CELSR3* | 0,26 | 0,42 | 0,54 | 0,18 |
| *DLG4* | 0,42 | 0,41 | 0,52 | 0,37 |
| *RYK* | 0,45 | 0,30 | 0,52 | 0,29 |
| *FZD3* | 0,45 | 0,30 | 0,51 | 0,05 |
| *SLC6A3* | 0,38 | 0,36 | 0,50 | 0,21 |
| *PARK7* | 0,15 | 0,16 | 0,48 | 0,09 |
| *NLGN2* | 0,02 | 0,44 | 0,46 | 0,03 |
| *GNAS* | -0,23 | 0,41 | 0,44 | 0,26 |
| *OPRK1* | -0,12 | 0,32 | 0,36 | 0,13 |
| *ITGB2* | -0,24 | 0,22 | 0,29 | 0,18 |
| *OR5T1* | 0,01 | 0,10 | -0,33 | 0,70 |
| *ADRA1A* | 0,16 | 0,17 | 0,18 | 0,66 |
| *OPRM1* | -0,55 | -0,03 | -0,16 | 0,65 |
| *SYT11* | -0,41 | 0,25 | 0,04 | 0,61 |
| *DNM2* | 0,17 | 0,07 | 0,47 | 0,60 |
| *MOXD1* | 0,15 | 0,02 | 0,25 | 0,60 |
| *SLC22A1* | -0,17 | 0,29 | -0,20 | 0,59 |
| *SYT4* | 0,34 | 0,04 | 0,22 | 0,58 |
| *DDC* | 0,22 | 0,49 | -0,19 | 0,57 |
| *GNG2* | 0,29 | 0,41 | 0,04 | 0,48 |
| *SYT9* | -0,19 | 0,19 | -0,28 | 0,45 |
| *SNCA* | 0,09 | 0,22 | 0,01 | 0,44 |
| *DRD3* | 0,09 | 0,11 | -0,02 | 0,40 |
| *HTR2A* | -0,19 | 0,37 | 0,06 | 0,40 |
| *CXCL12* | 0,27 | 0,33 | 0,27 | 0,36 |
| **Proportion of variance** | **37,71** | **15,16** | **7,84** | **3,56** |
